# Kaniadakis’s Information Geometry of Compositional Data

**DOI:** 10.3390/e25071107

**Published:** 2023-07-24

**Authors:** Giovanni Pistone, Muhammad Shoaib

**Affiliations:** 1 De Castro Statistics, Collegio Carlo Alberto, 10122 Torino, Italy; 2 Department of Mathematics, University of Genoa, 16144 Genova, Italy; muhammad.shoaib@dima.unige.it

**Keywords:** Kaniadakis logarithm, information geometry, compositional data, affine displacement, affine statistical bundle, barycenter, Kaniadakis divergence

## Abstract

We propose to use a particular case of Kaniadakis’ logarithm for the exploratory analysis of compositional data following the Aitchison approach. The affine information geometry derived from Kaniadakis’ logarithm provides a consistent setup for the geometric analysis of compositional data. Moreover, the affine setup suggests a rationale for choosing a specific divergence, which we name the Kaniadakis divergence.

## 1. Introduction

This paper describes Kaniadakis’ statistics as a methodology in data science. Precisely, we discuss Kaniadakis’ formalism for defining an affine structure on the open probability simplex. We present the methods in some generality and use them for the exploratory analysis of compositional data. The illustrating example is a small dataset, and we do not discuss any scaling issues of our methods. However, the dataset has an independent interest in financial risk analysis.

### 1.1. Why a Geometric Methodology

Kaniadakis’ logarithm [1,2] generalises the ordinary logarithm in a way that supports the development of deformed exponential families, deformed statistical divergences, and deformed entropy. Kaniadakis was originally motivated by the applications to non-extensive statistical physics in the sense of [3,4]. In this paper, we present the geometry of the probability simplex as a system of two affine spaces in duality from the perspective of information geometry (IG) [5]. The affine setup was first applied to deformed statistical models in [6].

The systematic use of this formal geometric perspective provides a robust and unified rationale for discussing key descriptive concepts. Defining geometry is much more than providing a topology or a distance. We provide a definition of affine geodesics and a natural duality so that the orthogonal surfaces of the geodesics are well-defined by a specific divergence function. The divergence level sets form a neighbourhood system and, eventually, a topology. In this setup, we define the barycentre, the displacement from the barycentre, and dimensionality reduction. For the standard affine geometry of the probability simplex, see, for example, the tutorial reference [7]. We use a special kind of Kaniadakis’ logarithm that appears with a different name in compositional data (CoDa) ([8] Example 4.20).

### 1.2. CoDa

Compositional data (or CoDa) are the data where all of a (row) vector’s (i.e., $\left[x_{1},x_{2},\ldots ,x_{D}\right]$) components are strictly positive real values, can also have zero values, and thus contain solely relative information; the composition is called a D-part composition. Compositional data are often expressed in closed form and totalled a fixed value, such as 1 for parts per unit or 100 for percentage measurements ([9] Chapter 2).

Compositional data are often found in geosciences and other scientific disciplines, and classification, discrimination, and categorization need to be adapted to the case of CoDa. CoDa analysis is closely related to geosciences and biology, where the data are mostly expressed as proportions or concentrations without mentioning the total size or amount explicitly [10].

Significant advancement has been accomplished during the last thirty to forty years. Recently, the term CoDa analysis has been employed to “Insist on the idea that the study goals or hypotheses, which place more of an emphasis on relative than absolute values, are what ultimately determine composition rather than the data, which may not be pieces of a whole or may not have a fixed sum” [11]. These qualities make CoDa analysis the most powerful tool for applications outside the tradition of hard sciences [12]. Current studies in management, economics, and social sciences have shown in practice the benefits of compositional methods in handling a wide variety of problems, which range from market shares and customer segmentation to tourism, transport systems, financial ratios, and many more (see [11,13,14,15]).

### 1.3. CoDa and Systemic Financial Risk

The Center for Risk Management at the University of Lausanne (http://www.crml.ch, accessed on 28 June 2023) provides systemic risk assessments for European financial institutions, which we used in our empirical study using the above Kaniadakis methods. The dataset enables the determination of SRISK country-level values, a market-based systemic risk indicator first proposed in [16,17] and most recently examined in [18].

The characteristics of SRISK are popular in the literature, and SRISK is mainly used to recognize weak institutions and countries with a system-wide impact before a crisis occurs [19] and can help forecast actual sector performance [20].

Most of the previous literature has mainly focused on the absolute values of SRISK. In this work, we focus on implementing the Kaniadakis methods to see the different European countries as a part of compositional data. We developed work started in [12], where they first introduced compositional data analysis to examine the distribution of relative contributions to SRISK connected with key European nations from 2008 to 2021.

Atchison [21] first introduced CoDa analysis. The research conducted by the [12] on financial data used the Atchison methods to examine how European nations contribute to the total amount of systemic risk (SRISK). They find that the distinctive quality of CoDa analysis, especially the Atchison geometry, is very effective in determining the threats of possible instability offered by smaller institutions and nations that might not completely emerge from the scale of their systemic risk.

### 1.4. Data and Methods

This paper first establishes a novel theoretical framework for compositional data using Kaniadakis’ logarithm. Second, we implement the Kaniadakis divergence on the compositional data and calculate the exponential and mixture displacements on compositional data. Next, we calculate the barycenter and deviation. The purpose of calculating the barycenter is to check how far the values of SRISK are from their centre value.

We consider ten European economies (Belgium, Denmark, France, Germany, Greece, Italy, Netherlands, Spain, Switzerland, and the UK) with annual SRISK measurements collected at the end of December for 2008–2021. Every number is stated in billions of Euros. Like most CoDa method applications, the sample does not cover Europe. Therefore, the ten components that make up our SRISK compositions are just a portion of all those that may be used. CoDa analysis, however, is predicated on the basic notion of sub-compositional coherence, which ensures that a compositional study conducted on a subset of components is consistent with the same analysis performed on the entire composition.

### 1.5. Kaniadakis’ Logarithm

We summarize the particular case of Kaniadakis’ logarithm with a purely algebraic form. In the suggestive formalism introduced by [22], the generalised logarithms are associated with the reciprocal derivative function *A*

(1) $$\begin{array}{c} \log_{\kappa }\left(x\right)=\frac{1}{2}\left(x-\frac{1}{x}\right)=\int_{1}^{x}\frac{du}{A(u)},\;with\;A\left(u\right)=\frac{2u^{2}}{1+u^{2}}\\ \exp_{\kappa }\left(y\right)=\log_{\kappa }^{-1}y=y+\sqrt{1+y^{2}}=\exp\int_{0}^{y}\frac{dv}{\sqrt{1+v^{2}}},\;x^{2}-2xy-1=0\;,\;x>0\;. \end{array}$$

Notice that the growth is linear in both directions.

Notice that the above equation reduces any polynomial in *y* and $x=\exp_{\kappa }\left(y\right)$, for example:

$$\begin{array}{c} \exp_{\kappa }{\left(y\right)}^{2}=2y\exp_{\kappa }\left(y\right)+1\;,\\ \exp_{\kappa }{\left(y\right)}^{3}=\exp_{\kappa }\left(y\right)\left(2y\exp_{\kappa }\left(y\right)+1\right)=\ldots , \end{array}$$

and so on. This is an algebraic feature, and this theory is a case of algebraic statistics [23].

The main known properties of the κ−logarithm and κ−exponential are

$$\begin{array}{c} \log_{\kappa }\left(\frac{1}{x}\right)=-\log_{\kappa }\left(x\right)\;,\;\exp_{\kappa }\left(y\right)\exp_{\kappa }\left(-y\right)=1\\ \frac{d}{dy}\exp_{\kappa }\left(y\right)=A\left(\exp_{\kappa }\left(y\right)\right)=\frac{1}{\sqrt{1+y^{2}}}\exp_{\kappa }\left(y\right) \end{array}$$

(2) $$\begin{array}{c} \exp_{\kappa }\left(x\right)\exp_{\kappa }\left(y\right)=\exp_{\kappa }\left(x\sqrt{1+y^{2}}+y\sqrt{1+x^{2}}\right) \end{array}$$

(3) $$\begin{array}{c} \log_{\kappa }\left(x_{2}\right)-\log_{\kappa }\left(x_{1}\right)<\frac{1}{A(x_{1})}\left(x_{2}-x_{1}\right)\;,\;x_{1}\neq x_{2} \end{array}$$

(4) $$\begin{array}{c} \exp_{\kappa }\left(y_{2}\right)-\exp_{\kappa }\left(y_{1}\right)>A\left(\exp_{\kappa }\left(y_{1}\right)\right)\left(y_{2}-y_{1}\right)\;,\;y_{1}\neq y_{2} \end{array}$$

### 1.6. Kaniadakis’ Exponential Form of a Positive Probability Function

If the sample space Ω is a finite set, then the probability simplex on Ω is $P\left(\Omega \right)$, and the open probability simplex is $Ԑ\left(\Omega \right)$.

For all $p\in Ԑ\left(\Omega \right)$, the function A∘p, *A* as in Equation (1), is strictly positive and provides a positive weight on Ω. It is proportional, but usually not equal, to a probability function,

$$\tilde{p}\propto A\circ p\;,\;\mathrm{namely},\;\tilde{p}\left(x\right)=\frac{A(p(x))}{\sum_{x\in \Omega }A\left(p\left(x\right)\right)}\;.$$

We will also write $\tilde{p}=A\circ p/\bar{A\circ p}$. The mapping $Ԑ\left(\Omega \right)∋p\mapsto \tilde{p}\in Ԑ\left(\Omega \right)$ is called the *escort* mapping; see [22]. See ([24] §3.1) for a discussion of its injectivity and surjectivity. We introduce a notation for the escort expectation, ${\tilde{𝔼}}_{p}\left[u\right]=\sum_{x\in \Omega }u\left(x\right)\tilde{p}\left(x\right)$.

For $p,q\in Ԑ\left(\Omega \right)$, the *Kaniadakis divergence* can be defined by changes in the usual definition of the logarithm to the Kaniadakis logarithm and the probability function *p* with the escort $\tilde{p}$:

(5) $$\begin{array}{cc} \tilde{D}\left(p|q\right) & ={\tilde{𝔼}}_{p}\left[\log_{\kappa }\left(p\right)-\log_{\kappa }\left(q\right)\right]\\ \; & =\sum_{x\in \Omega }\left(\log_{\kappa }\left(p\right)-\log_{\kappa }\left(q\right)\right)\;A\circ p/\bar{A\circ p}\\ \; & =\sum_{x\in \Omega }\left(\log_{\kappa }\left(p\right)-\log_{\kappa }\left(q\right)\right)\tilde{p}\left(x\right) \end{array}$$

Clearly, $\tilde{D}\left(p|p\right)=0$. If p≠q, from the concavity in Equation (3),

$$\begin{array}{cc} \tilde{D}\left(p|q\right) & ={\left(\bar{A\circ p}\right)}^{-1}\sum_{x\in \Omega }A\left(p\left(x\right)\right)\left(\log_{\kappa }\left(p(x)\right)-\log_{\kappa }\left(q(x)\right)\right)\\ \; & >{\left(\bar{A\circ p}\right)}^{-1}\sum_{x\in \Omega }\left(p\left(x\right)-q\left(x\right)\right)\\ & =0\;. \end{array}$$

Fix $p\in Ԑ\left(\Omega \right)$. For all $q\in Ԑ\left(\Omega \right)$, define

$$s_{p}\left(q\right)=\left(\log_{\kappa }\left(q\right)-\log_{\kappa }\left(p\right)\right)+\tilde{D}\left(p|q\right)=\left(\log_{\kappa }\left(q\right)-\log_{\kappa }\left(p\right)\right)-{\tilde{𝔼}}_{p}\left[(\log_{\kappa }\left(q\right)-\log_{\kappa }\left(p\right))\right]\;,$$

then, for $u=s_{p}\left(q\right)$,

(6) $$q=\exp_{\kappa }\left(u-\tilde{D}\left(p|q\right)+\log_{\kappa }\left(p\right)\right)\;,\;{\tilde{𝔼}}_{p}\left[u\right]=0\;.$$

Conversely, for all $p\in Ԑ\left(\Omega \right)$, if *u* is a random variable such that ${\tilde{𝔼}}_{p}\left[u\right]=0$, the real function

$$R_{+}∋\psi \mapsto \sum_{x\in \Omega }\exp_{\kappa }\left(u\left(x\right)-\psi +\log_{\kappa }\left(p(x)\right)\right)$$

is continuous, goes to 0 as ψ→∞, and, for ψ=0, takes a value larger than 1 because of Equation (4):

$$\begin{array}{cc} \sum_{x\in \Omega }\exp_{\kappa }\left(u\left(x\right)+\log_{\kappa }\left(p(x)\right)\right) & >\sum_{x\in \Omega }\exp_{\kappa }\left(\log_{\kappa }\left(p(x)\right)\right)+\sum_{x\in \Omega }A(\exp_{\kappa }\left(\log_{\kappa }\left(p(x)\right)\right)u\left(x\right)\\ \; & =\sum_{x\in \Omega }p\left(x\right)+\sum_{x\in \Omega }A\left(p\left(x\right)\right)u\left(x\right)\\ \; & =1\;. \end{array}$$

In conclusion, there exists a function,

$$K_{p}:S_{p}=\left\{u\in L\left(\Omega \right)\;|\;{\tilde{𝔼}}_{p}\left[u\right]=0\right\}∋u\mapsto K_{p}\left(u\right)\geq 0\;,$$

and $K_{p}\left(u\right)>0$ provided u≠0, such that

$$q=\exp_{\kappa }\left(u-K_{p}\left(u\right)+\log_{\kappa }\left(p\right)\right)\in Ԑ\left(\Omega \right)\;.$$

Hence, we have

$$K_{p}\left(u\right)=\tilde{D}\left(p|q\right)$$

and the mapping

$$s_{p}:Ԑ\left(\Omega \right)\to S_{p}$$

is a bijection with inverse

(7) $$e_{p}:S_{p}∋u\mapsto \exp_{\kappa }\left(u-K_{p}\left(u\right)+\log_{\kappa }\left(p\right)\right)\in Ԑ\left(\Omega \right)\;.$$

### 1.7. Properties of the Cumulant Function $K_{p}$

Let us compute the derivatives of the function $K_{p}$. We use a square bracket notation for the direction

$$dK_{p}\left(u\right)\left[h\right]=\lim_{\theta \to 0}\theta^{-1}\left(K_{p}\left(u+\theta h\right)-K_{p}\left(u\right)\right)={\left.\frac{d}{d\theta }K_{p}\left(u+\theta h\right)\right|}_{\theta =0}\;.$$

From Equation (7),

$$\begin{array}{cc} 0 & ={\left.\frac{d}{d\theta }\sum_{x\in \Omega }\exp_{\kappa }\left(u\left(x\right)+\theta h\left(x\right)-K_{p}\left(u+\theta h\right)+\log_{\kappa }\left(p(x)\right)\right)\right|}_{\theta =0}\\ \; & ={\left.\sum_{x\in \Omega }A\left(\exp_{\kappa }\left(u\left(x\right)+\theta h\left(x\right)-K_{p}\left(u+\theta h\right)+\log_{\kappa }\left(p(x)\right)\right)\right)\left(h\left(x\right)-\frac{d}{dt}K_{p}\left(u+\theta h\right)\right)\right|}_{\theta =0}\\ \; & =\sum_{x\in \Omega }A\left(\exp_{\kappa }\left(u\left(x\right)-K_{p}\left(u\right)+\log_{\kappa }\left(p(x)\right)\right)\right)\left(h\left(x\right)-dK_{p}\left(u\right)\left[h\right]\right)\\ \; & =\sum_{x\in \Omega }A\left(e_{p}\left(u\right)\right)\left(h-dK_{p}\left(u\right)\left[h\right]\right)\;. \end{array}$$

It follows that, for each $p\in Ԑ\left(\Omega \right)$ and $u,h\in S_{p}$, it holds

(8) $$dK_{p}\left(u\right)\left[h\right]={\tilde{𝔼}}_{q}\left[h\right]\;,$$

where $q=e_{p}\left(u\right)$; see Equation (7).

If the curve t↦q(t) has constant divergence, that is, $\tilde{D}\left(p|q(t)\right)=\tilde{D}\left(p|q(0)\right)$, derivation provides

$$0=\frac{d}{dt}\tilde{D}\left(p|q(t)\right)=\frac{d}{dt}K_{p}\left(u\left(t\right)\right)=dK_{p}\left(u\left(t\right)\right)\left[\dot{u}\left(t\right)\right]={\tilde{𝔼}}_{q(t)}\left[\dot{u}\left(t\right)\right]\;.$$

Notice that ${\tilde{𝔼}}_{q(0)}\left[u(t)\right]=0$, but this does not imply ${\tilde{𝔼}}_{q(t)}\left[\dot{u}\left(t\right)\right]$ unless the previous conditions hold true.

### 1.8. Bibliographical Notes

Similarly, $d^{2}K_{p}$ and the convex conjugate of $K_{p}$ can be computed. See below for the duality and see also [7,25]. Kaniadakis logarithm and exponential were first introduced in [26,27]. The application to IG used here appeared in [6,24,28]. These papers discuss both the finite state space and the general state space.

## 2. Affine Space

The Kaniadakis non-parametric affine geometry of the open probability simplex is a variation of the standard case [7]. The main difference is the substitution of the expectation with the escort expectation.

### 2.1. Statistical Bundle

The statistical bundle is an expression of the tangent space of $Ԑ\left(\Omega \right)$ as a dually flat affine statistical manifold in the sense of [5]. The statistical bundle $SԐ\left(\Omega \right)$ and each fiber $S_{q}Ԑ\left(\Omega \right)$ are defined by

(9) $$\begin{array}{c} SԐ\left(\Omega \right)=\left\{\left(q,v\right)\;|\;q\in Ԑ\left(\Omega \right),{\tilde{𝔼}}_{q}\left[v\right]=0\right\}\;, \end{array}$$

(10) $$\begin{array}{c} S_{q}Ԑ\left(\Omega \right)=\left\{v\in L\left(\Omega \right)\;|\;{\tilde{𝔼}}_{q}\left[u\right]=0\right\}\;,\;q\in Ԑ\left(\Omega \right)\;. \end{array}$$

In our setup, each fibre is a finite-dimensional vector space and can be identified with its dual. However, it is convenient to distinguish the two statistical bundles. The previous one is called *exponential* statistical bundle, while the *mixture* statistical bundle is

(11) S*ԐΩ=(q,v)|q∈ԐΩ,𝔼˜qv=0,

(12) S*qԐΩ=v∈L(Ω)|𝔼˜qu=0,q∈ԐΩ.

For each couple $p,q\in Ԑ\left(\Omega \right)$, the mapping

(13) Uepq:SpԐΩ∋v↦v−𝔼˜qv∈SqԐΩ

is a bijection. The Uepp is the identity mapping, and

UeqrUepq=Uepr.

The co-cycle of mappings (Uepq)p,q is the *exponential parallel transport* of the exponential statistical bundle.

The mapping defined for all $p\in Ԑ\left(\Omega \right)$, v∈S*pԐΩ, and $w\in S_{p}Ԑ\left(\Omega \right)$ by

$$g:\left(p,v,w\right)\mapsto g_{p}\left(v,w\right)={\langle v,w\rangle }_{A\circ p}=\sum_{x\in \Omega }A\left(p\left(x\right)\right)u\left(x\right)v\left(x\right)=\bar{A\circ p}\;{\tilde{𝔼}}_{p}\left[vw\right]$$

provides a duality between the fibres of $SԐ\left(\Omega \right)$ and S*ԐΩ.

The dual of the exponential transport can be computed as follows. For $p,q\in Ԑ\left(\Omega \right)$, v∈S*qԐΩ, and $w\in S_{p}Ԑ\left(\Omega \right)$,

v,UepqwA∘q=∑x∈ΩA∘qv(w−𝔼˜qw)=∑x∈ΩA∘qvw−𝔼˜qw∑A∘qv=∑x∈ΩA∘qvw=∑x∈ΩA∘pA∘qA∘puw=A∘qA∘pu,wA∘p.

Now, A∘qA∘pv∈S*pԐΩ; hence, the dual of the exponential transport is the *mixture transport*,

(14) Ueqp*v=Umqpv=A∘qA∘pv.

### 2.2. Velocity and Auto-Parallel Curves

The following computation is a version of the original argument about Fisher’s score. Let $t\mapsto q\left(t\right)\in Ԑ\left(\Omega \right)$ be a one-dimensional parametric statistical model, namely a curve in geometric language. We assume the curve is smooth and twice differentiable as a mapping in the vector space L(Ω). For each random variable f∈L(Ω),

(15) $$\begin{array}{cc} \frac{d}{dt}E_{q(t)}\left[f\right] & =\sum_{x\in \Omega }f\left(x\right)\dot{q}\left(x;t\right)\\ \; & =\sum_{x\in \Omega }f\left(x\right)\frac{\dot{q}\left(x;t\right)}{A(q(x;t))}A\left(q\left(x;t\right)\right)\\ \; & =\sum_{x\in \Omega }f\left(x\right)\frac{d}{dt}\log_{\kappa }\left(q(x;t\right)A\left(q\left(x;t\right)\right)\\ \; & =\sum_{x\in \Omega }A\circ q\left(t\right)\left(f-E_{\tilde{q}\left(t\right)}\left[f\right]\right)\frac{d}{dt}\log_{\kappa }\left(q(t)\right)\\ \; & ={\langle f-E_{\tilde{q}\left(t\right)}\left[f\right],\frac{d}{dt}\log_{\kappa }\left(q(t)\right)\rangle }_{A\circ q(t)}\\ \; & =\sum_{x\in \Omega }f\left(x\right)\dot{q}\left(x;t\right)\\ \; & ={\langle f-E_{\tilde{q}\left(t\right)}\left[f\right],\frac{d}{dt}\log_{\kappa }\left(q(t)\right)\rangle }_{A\circ q(t)} \end{array}$$

The *velocity* of the curve is defined as

(16) $$\overset{★}{q}\left(t\right)=\frac{d}{dt}\log_{\kappa }\left(q(t)\right)=\frac{\dot{q}\left(t\right)}{A(q(t))}\;.$$

We can check that $\overset{★}{q}\left(t\right)\in S_{q(t)}Ԑ\left(\Omega \right)$ and (f−Eq˜(t)f)∈S*q(t)ԐΩ. The *Cramer–Rao bound* is

(17) $$\begin{array}{cc} {\left(\frac{d}{dt}E_{q(t)}\left[f\right]\right)}^{2}\; & ={\bar{A\circ q(t)}}^{2}{\langle f-E_{\tilde{q}\left(t\right)}\left[f\right],\frac{d}{dt}\log_{\kappa }\left(q(t)\right)\rangle }_{\tilde{q}\left(t\right)}\\ \; & \leq {\bar{A\circ q(t)}}^{2}E_{\tilde{q}\left(t\right)}\left[{\left(f-E_{\tilde{q}\left(t\right)}\left[f\right]\right)}^{2}\right]E_{\tilde{q}\left(t\right)}\left[{\left(\frac{\dot{q}\left(t\right)}{A\circ q(t)}\right)}^{2}\right]\\ \; & =\sum_{x\in \Omega }A\circ q\left(t\right){\left(f-{\tilde{𝔼}}_{q}\left[f\right]\right)}^{2}\;\sum \frac{\dot{q}{\left(t\right)}^{2}}{A\circ q(t)}\\ \; & =\sum_{x\in \Omega }A\circ q\left(t\right){\left(f-{\tilde{𝔼}}_{q}\left[f\right]\right)}^{2}\;\sum \frac{\dot{q}{\left(t\right)}^{2}}{A\circ q(t)} \end{array}$$

The variation computed with the escort probability function, namely $f-{\tilde{𝔼}}_{q}\left[f\right]$, appears in Equation (15) as a gradient of the expectation $f\mapsto E_{q}\left[f\right]$.

A curve t↦q(t) is *auto-parallel* for the mixture trasport if

Umq(t)q(s)q★(t)=q★(s).

For $q\left(0\right)=q_{0}$ and $q\left(1\right)=q_{1}$,

$$\frac{\dot{q}\left(0\right)}{A\circ q_{0}}=\frac{A\circ q(t)}{A\circ q_{0}}\frac{\dot{q}\left(t\right)}{A\circ q(t)}=\frac{1}{A\circ q_{0}}\dot{q}\left(t\right)\;,$$

so that

$$q\left(t\right)=q_{0}+t\left(q_{1}-q_{0}\right)\;.$$

Let us compute the auto-parallel curves for the exponential transport,

Ueq(t)q(s)q★(t)=q★(s).

For $q\left(0\right)=q_{0}$ and $q\left(1\right)=q_{1}$,

$$\overset{★}{q}\left(0\right)=\overset{★}{q}\left(t\right)-{\tilde{𝔼}}_{q_{0}}\left[\overset{★}{q}\left(t\right)\right]=\frac{d}{dt}\log_{\kappa }\left(q(t)\right)-\frac{1}{\bar{A\circ q_{0}}}\sum A\circ q_{0}\frac{d}{dt}\log_{\kappa }\left(q(t)\right)\;,$$

so that, for some function ψ,

$$\log_{\kappa }\left(q(t)\right)=\log_{\kappa }\left(q_{0}\right)+t\overset{★}{q}\left(0\right)-\psi \left(t\right)\;.$$

Comparing with Equation (7), we have that *the auto-parallel curve for the exponential transport is*

(18) $$q\left(t\right)=\exp_{\kappa }\left(t\overset{★}{q}\left(0\right)-K_{q_{0}}\left(t\overset{★}{q}\left(0\right)\right)+\log_{\kappa }\left(q_{0}\right)\right)\;.$$

As observed above,

$$\overset{★}{q}\left(0\right)=\log_{\kappa }\left(q_{1}\right)-\log_{\kappa }\left(q_{0}\right)-{\tilde{𝔼}}_{q_{0}}\left[\log_{\kappa }\left(q_{1}\right)-\log_{\kappa }\left(q_{0}\right)\right]=\log_{\kappa }\left(q_{0}\right)+\tilde{D}\left(q_{0}|q_{1}\right)\;.$$

### 2.3. Surfaces of Constant Divergence

We have observed that an auto-parallel curve starting at q(0) with velocity $\overset{★}{q}\left(0\right)$ has the form of Equation (18). For given extreme points $q_{0}=q\left(0\right)$ and $q_{1}=q\left(1\right)$, it holds that

$$q_{1}=\exp_{\kappa }\left(u-K_{q_{0}}\left(u\right)+\log_{\kappa }\left(q_{0}\right)\right)\;\mathrm{hence}\;u=\log_{\kappa }\left(q(0)\right)-\log_{\kappa }\left(q(1)\right)+\tilde{D}\left(q_{0}|q_{1}\right)\;,$$

in particular, ${\tilde{𝔼}}_{q_{1}}\left[u\right]=0$.

The velocity of the auto-parallel curve at $q_{1}$ is constant,

$${\left.\frac{d}{dt}\left(tu-K_{q_{0}}\left(tu\right)+\log_{\kappa }\left(q_{0}\right)\right)\right|}_{t=1}=u-{\tilde{𝔼}}_{q_{1}}\left[u\right]=u.$$

Consider a curve γ starting at $\gamma \left(0\right)=q_{1}$ of the form,

$$t\mapsto \gamma \left(t\right)=\exp_{\kappa }\left(u+v\left(t\right)-K_{q_{0}}\left(u+v\left(t\right)\right)+\log_{\kappa }\left(q_{0}\right)\right)\;\mathrm{with}\;v(0)=0$$

and assume a divergence is constant, precisely

$$\tilde{D}\left(\gamma \left(t\right)|q_{0}\right)=\tilde{D}\left(\gamma \left(0\right)|q_{0}\right)=\tilde{D}\left(q_{1}|q_{0}\right)\;.$$

It holds

$$\begin{array}{cc} {\tilde{𝔼}}_{\gamma (t)}\left[\log_{\kappa }\left(\gamma (t)\right)-\log_{\kappa }\left(q_{0}\right)\right] & ={\tilde{𝔼}}_{\gamma (t)}\left[u+v\left(t\right)-K_{q_{0}}\left(u+v\left(t\right)\right)+\log_{\kappa }\left(q_{0}\right)-\log_{\kappa }\left(q_{0}\right)\right]\\ & ={\tilde{𝔼}}_{\gamma (t)}\left[u+v(t)\right]-K_{q_{0}}\left(u+v\left(t\right)\right)\\ \; & =dK_{q(0)}\left(u+v\left(t\right)\right)\left[u+v\left(t\right)\right]-K_{q_{0}}\left(u+v\left(t\right)\right) \end{array}$$

is constant so that the derivative is zero. In particular, it is zero at t=0

$$0={\left.d^{2}K_{q_{0}}\left[u+v\left(t\right)\right]\left[u+v\left(t\right),\dot{v}\left(t\right)\right]\right|}_{t=0}=d^{2}K_{q_{0}}\left[u,\dot{v}\left(0\right)\right]\;.$$

That is, this surface of equi-divergence is orthogonal to the auto-parallel curves in the sense of the quadratic form $d^{2}K$. This is actually the generalization of a well-known result in IG, where the Hessian of the cumulant function is the Fisher’s information matrix. See, for example, [5].

### 2.4. Displacement

The machinery introduced above allows for explicitly defining the affine structure as originally defined by [29]. A textbook on affine geometry is ([30] Ch. 2,3,9). Below, we call the following two (dual) displacements on the statistical bundle. The mixture displacement is

(19) $$\eta_{p}\left(q\right)=\frac{q-p}{A\circ p}\;.$$

The exponential displacement is

(20) $$s_{p}\left(q\right)=\left(\log_{\kappa }\left(q\right)-\log_{\kappa }\left(p\right)\right)-{\tilde{𝔼}}_{p}\left[\left(\log_{\kappa }\left(q\right)-\log_{\kappa }\left(p\right)\right)\right]$$

Both displacements define affine coordinates in the statistical bundle. The easy proofs are the same as in the standard cases [7]. Each displacement defines an atlas of charts on the affine bundle.

The orthogonal surfaces of the affine exponential auto-parallel curves are discussed in the section above. The orthogonal surfaces to the affine mixture auto-parallel curves are easily observed to be associated with the other divergence. In fact, it is the classical result of the duality between the two divergences. See, for example, [5].

The availability of an affine bundle would allow for a coherent and straightforward definition of mechanical concepts such as velocity, acceleration, Lagrangian, and Hamiltonian. See [31,32] for the standard case. In the present paper, we develop the application to CoDa, and we stress the notion of affine barycenter and the fact that a system of charts can be observed as a preprocessing of data to be followed by any method adapted to actual vector data.

### 2.5. Barycenter and Deviation

Let $f_{1},\ldots ,f_{n}$ be a sequence of CoDa points with strictly positive components and normalized to one. Each data point is a point in the open probability simplex. The affine coordinates (20) centered at *p* are

$$\begin{array}{cc} s_{p}\left(f_{1}\right) & =\left(\log_{\kappa }\left(f_{1}\right)-\log_{\kappa }\left(p\right)\right)-{\tilde{𝔼}}_{p}\left[\left(\log_{\kappa }\left(f_{1}\right)-\log_{\kappa }\left(p\right)\right)\right]\\ \vdots \\ s_{p}\left(f_{n}\right) & =\left(\log_{\kappa }\left(f_{n}\right)-\log_{\kappa }\left(p\right)\right)-{\tilde{𝔼}}_{p}\left[\left(\log_{\kappa }\left(f_{n}\right)-\log_{\kappa }\left(p\right)\right)\right] \end{array}$$

The *mean value* of the affine coordinates is

(21) $$\begin{array}{cc} \bar{s_{p}} & =\frac{1}{n}\sum_{j=1}^{n}s_{p}\left(f_{j}\right)=\frac{1}{n}\sum_{j=1}^{n}\left(\log_{\kappa }\left(f_{j}\right)-{\tilde{𝔼}}_{p}\left[\left(\log_{\kappa }\left(f_{j}\right)\right)\right]\right)-\left(\log_{\kappa }\left(p\right)-{\tilde{𝔼}}_{p}\left[\log_{\kappa }\left(p\right)\right]\right) \end{array}$$

If the mean value computed in the centering *q* is $\bar{s_{q}}$, the difference is

$$\bar{s_{p}}-\bar{s_{q}}=\log_{\kappa }\left(q\right)-\log_{\kappa }\left(p\right)-\frac{1}{n}\sum_{j=1}^{n}{\tilde{𝔼}}_{p}\left[\log_{\kappa }\left(f_{j}\right)\right]+\frac{1}{n}\sum_{j=1}^{n}{\tilde{𝔼}}_{q}\left[\log_{\kappa }\left(f_{j}\right)\right]\;.$$

Hence,

$$\bar{s_{p}}+\log_{\kappa }\left(p\right)=\bar{s_{q}}+\log_{\kappa }\left(q\right)+\mathrm{constant}\;.$$

The probability function is the same in both cases. In fact,

$$\begin{array}{cc} \exp_{\kappa }\left(\bar{s_{p}}-K_{p}\left(\bar{s_{p}}\right)+\log_{\kappa }\left(p\right)\right) & =\exp_{\kappa }\left(\bar{s_{q}}-K_{p}\left(\bar{s_{p}}\right)+\log_{\kappa }\left(q\right)+\mathrm{constant}\right)\\ \; & =\exp_{\kappa }\left(\bar{s_{q}}-K_{q}\left(\bar{s_{q}}\right)+\log_{\kappa }\left(q\right)\right) \end{array}$$

because of the uniqueness of the normalizing constant.

In conclusion, the probability function

$$\begin{array}{cc} \bar{f} & =\exp_{\kappa }\left(\bar{s_{p}}-K_{p}\left(\bar{s_{p}}\right)+\log_{\kappa }\left(p\right)\right)\\ \; & =\exp_{\kappa }\left(\bar{s_{p}}+\tilde{D}\left(p|\bar{f}\right)+\log_{\kappa }\left(p\right)\right)\;, \end{array}$$

with $\bar{s_{p}}$ as Equation (21) *does not depend on the reference p*. It is the *barycentre* of the given data points.

The displacement of each data point $f_{j}$ from the barycentre $\bar{f}$ is

$$\begin{array}{cc} s_{\bar{f}}\left(f_{j}\right)=\bar{s_{p}}-s_{p}\left(f_{j}\right)-{\tilde{𝔼}}_{\bar{f}}\left[\bar{s_{p}}-s_{p}\left(f_{j}\right)\right] & =\frac{1}{n}\sum_{k}s_{p}\left(f_{k}\right)-s_{p}\left(f_{j}\right)-{\tilde{𝔼}}_{\bar{f}}\left[\frac{1}{n}\sum_{k}s_{p}\left(f_{k}\right)-s_{p}\left(f_{j}\right)\right] \end{array}$$

and the expression of each point $f_{j}$ in the barycentre $\bar{f}$ is

$$f_{j}=\exp_{\kappa }\left(s_{\bar{f}}\left(f_{j}\right)+\tilde{D}\left(\bar{f}|f_{j}\right)+\log_{\kappa }\left(\bar{f}\right)\right)\;.$$

A one-dimensional summary consistent with our formalism of the divergence of each point from the barycentre is the Kaniadakis’ divergence $\tilde{D}\left(\bar{f}|f_{j}\right)$, which is the normalising constant in the equation above. Another option is the Kaniadakis’ divergence $\tilde{D}\left(f_{j}|\bar{f}\right)$ that appears in the representation of the barycentre in the data point $f_{j}$.

## 3. Data Analysis

This section will use some geometric concepts derived from Kaniadakis’ IG. It should be noted that our formalism is, in principle, affine and does not include any properly defined distance.

### 3.1. Kaniadakis Divergence

**Figure 1:**
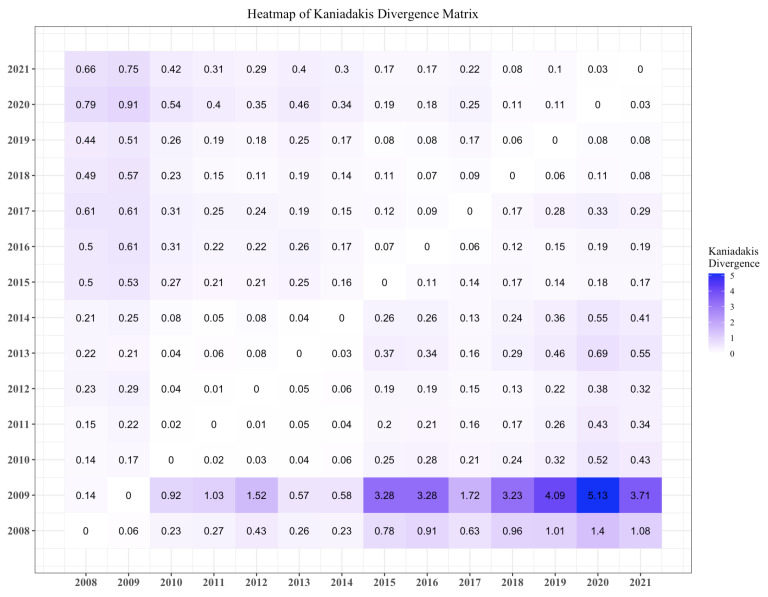
Kaniadakis divergence on compositional data.

First, we compute the Kaniadakis divergence defined in Equation (5). Each point in (i,j) in Figure 1 is the Kaniadakis divergence of the CoDa point corresponding to the year in the *i*-th row with respect to the CoDa point for the year in the *j*-th column. For example, the Kaniadakis divergence between 2008 and 2009 is $\tilde{D}\left(2008|2009\right)=0.14$. Most values are smaller than one, except when the reference distribution corresponds to 2008 or 2009 for the most recent years. The year 2009 deviates significantly from the other years.

### 3.2. Mixture Displacement

**Figure 2:**
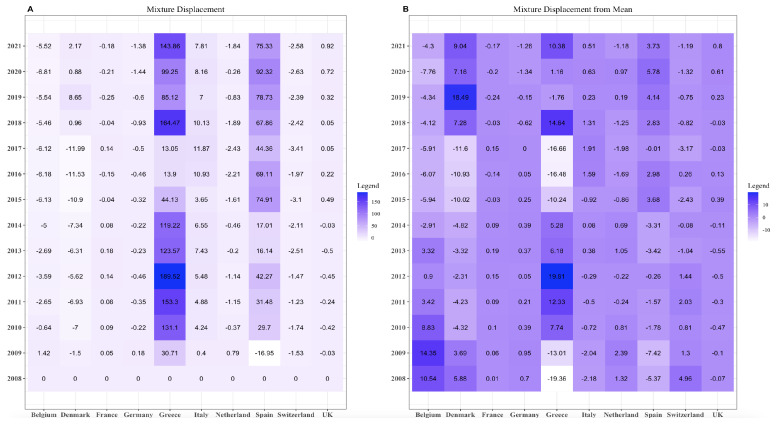
(**A**) Mixture displacement on compositional data by taking 2008 as a reference and (**B**) mixture displacement on compositional data by taking mean as reference.

Equation (19) provides instructions for computing the mixture displacement. From Figure 2, the mixture displacement for Greece and Spain is very high for all the years. The value for Spain in 2009 was less than zero—the only negative value for Spain. On the contrary, all other countries do not have too many high values.

Equation (21) provides the mean value. After determining the mean, we compute the mixture displacement using the mean as a reference. We check that our values abruptly go from −10 to 10. However, the results for Greece and Spain decrease when the mixture displacement from the mean is calculated.

### 3.3. Exponential Displacement

**Figure 3:**
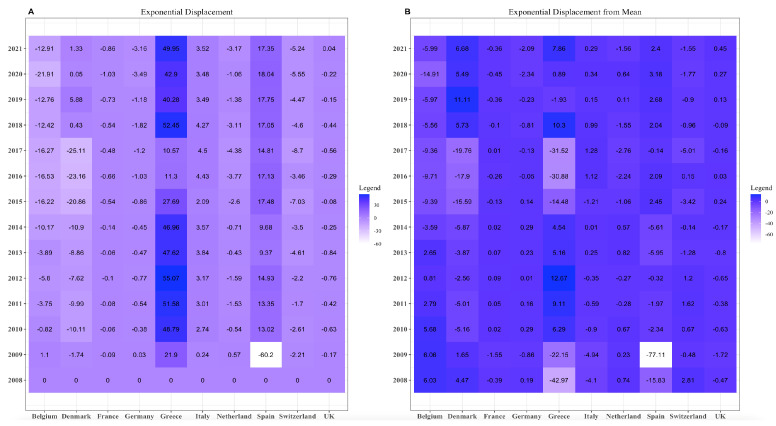
(**A**) Exponential displacement on compositional data by taking 2008 as a reference and (**B**) exponential displacement on compositional data by taking mean as a reference.

As above, Equation (20) returns the exponential displacement. Further, Figure 3 is the empirical result of Equation (20). As for the mixture displacement, we can see that Spain and Greece have higher displacement than other European countries. The value for Spain in the year 2009 is meagre.

If the mean is the reference point, the exponential displacement ranges from 0 to −60. The only significant changes are for the nations of Spain and Greece, where our values for 2009 for Spain decreased by about 18 times, and, for Greece, our values decreased significantly.

## 4. Conclusions and Discussion

In this research, we applied a particular type of divergence, Kaniadakis divergence, to compositional data, aligned with the symmetrised ratio transformation in ([8] Example 4.20). The dataset being examined spans the years 2008 through 2021. First, we built a theoretical framework for Kaniadakis divergence, mixture displacement, and exponential displacement.

Section 1 provided the mathematical framework for determining divergence and displacement, while Section 2 demonstrated how to apply those mathematical algorithms to compositional data. In the application, we found that Spain and Greece have more fluctuations when compared to the other European countries. The values of the mixture and exponential displacement confirm that Spain and Greece faced some financial crises compared to other countries.

This simple application shows the potential of IG for application to compositional data analysis. We suggest that Kaniadakis’ logarithm can reduce the computations for monitoring systemic risk to algebraic computations. The Kaniadakis logarithm, mixture, and exponential displacement on compositional data can be considered to broaden traditional research methods for compositional data analysis.

We would like to add a few words regarding the specific tools and formalism we used here. First, we mimicked one of the possible presentations of non-parametric IG by following the basic dually-flat setup step by step. Another successful presentation of non-parametric IG starts with properly defining the divergences and deriving the geometry; see, for example, [33]. A popular approach, not equivalent to the affine one, defines the geometry of the probability simplex by introducing a metric tensor. As in other geometric theories, one should carefully distinguish between choosing charts and introducing a topology.

In the present approach, we define the charts so that the associated manifold is affine; in this setup, some specific divergences appear as naturally associated with the geometry and the basic statistical notion, namely the pairing between measures and random variables. Everything is applied to simple data manipulation in the spirit of Aitchison’s methods.

No claim of optimality is made. The existence of many different but topologically equivalent divergences is only natural in our setup, where the topology actually depends on the geometry and not the other way around. Whenever needed, a choice must be based on some additional assumption. We carefully check the simple, useful operations on data, such that the geodesic connecting two given points, the velocity of variation, the barycentre, and the deviation from the barycentre are all defined correctly.

## Data Availability

Data are publicly available on the website (http://www.crml.ch, accessed on 1 May 2023).

## Abbreviations

CoDa	Compositional Data
SRISK	Systemic Risk
IG	Information Geometry
